# Application of Empirical Mode Decomposition with Local Linear Quantile Regression in Financial Time Series Forecasting

**DOI:** 10.1155/2014/708918

**Published:** 2014-07-22

**Authors:** Abobaker M. Jaber, Mohd Tahir Ismail, Alsaidi M. Altaher

**Affiliations:** ^1^School of Mathematical Sciences, Universiti Sains Malaysia, 11800 Minden, Penang, Malaysia; ^2^Statistics Department, Sebha University, Sebha 00218, Libya

## Abstract

This paper mainly forecasts the daily closing price of stock markets. We propose a two-stage technique that combines the empirical mode decomposition (EMD) with nonparametric methods of local linear quantile (LLQ). We use the proposed technique, EMD-LLQ, to forecast two stock index time series. Detailed experiments are implemented for the proposed method, in which EMD-LPQ, EMD, and Holt-Winter methods are compared. The proposed EMD-LPQ model is determined to be superior to the EMD and Holt-Winter methods in predicting the stock closing prices.

## 1. Introduction

Recent studies have indicated that financial markets typically follow nonlinear and nonstationary behavior. Therefore, forecasting finance using classical techniques is quite difficult. The empirical mode decomposition (EMD) explored by Huang et al. [1] is a very powerful tool in modern quantitative finance and has emerged as a powerful statistical modeling technique [2, 3]. The capacity of the EMD to handle nonlinear and nonstationary behaviors has provided both researchers and practitioners with an attractive alternative tool. The EMD can explain the generation of time series data from an alternative perspective by breaking up time series signals into smaller numbers of independent and concretely implicational intrinsic modes based on scale separation. This distinguishing feature makes the EMD a valuable and desirable tool for forecasting financial time series signals [4]. The current study aims to extract and forecast the trend of two stock markets, namely, the Kuala Lumpur Bursa (KLSE) index and the New Zealand stock market index (NZX50), using the advantages of local linear quantile (LLQ) regression. The proposed method consists of two stages. In the first stage, LLQ is applied to corrupt and noisy data. The remaining series is subsequently expected to be hidden in the residuals. In the second stage, EMD is applied to the residuals. The final estimate is the summation of the fitting estimates from the LLQ and EMD. To extract and forecast the trend using EMD-LLQ and EMD, we summarize the steps as follows. (1) A signal is decomposed by the EMD-LLQ and EMD. (2) Meaningful intrinsic mode functions (IMFs) (components) are selected using the fast Fourier transform (FFT) (see [5]). (3) Selected components are added to the residue to obtain the trend. (4) The Holt-Winter method is based on the selected components and provides the forecasting results.

The remainder of this paper is organized as follows.  Section 2 presents a brief background of the EMD and LLQ.  Section 3 introduces the proposed method.  Section 4 compares the results of the original EMD algorithm and the new proposed method by forecasting the daily closing prices of two stock markets, namely, the KLSE and the NZX50.  Section 5 concludes.

## 2. Why EMD-LPQ (Empirical Mode Decomposition Combined with Local Linear Quantile Regression)?

Due to the edged effects, nonparametric techniques such as empirical mode decomposition show a sharp increase in variance and bias at points near the boundary. The presence of such problem has dramatic effects on results. Varieties of works have been reported in literature in order to reduce the effects of boundary problem in traditional EMD. Two-stage methods or coupling methods nowadays have been used extensively for solving such problem; for instance [6], applied neural network to each IMF to restrain the end effect [7] provided an algorithm based on the sigma-pi neural network which is used to extend signals before applying EMD. Reference [4] proposed a new two-stage algorithm. The algorithm includes two steps: the extrapolation of the signal through support vector (SV) regression at both endpoints to form the primary expansion signal, and then the primary signal is further expanded through extrema mirror expansion and EMD is performed on the resulting signal to obtain reduced end limitations. All previous methods have been shown to have good solutions to the end point and achieved a higher precision in an application part as well. In this paper we have followed [8]. The proposed method EMD-LPQ (empirical mode decomposition combined with local linear quantile regression) is designed to be a robust version of classical empirical mode decomposition especially in presence of edge effect problems.

## 3. Empirical Mode Decomposition

The EMD [1] has proven to be a natural extension of and an alternative technique to traditional methods for analyzing nonlinear and nonstationary signals, such as wavelet methods, Fourier methods, and empirical orthogonal functions [9]. In this section, we briefly describe the EMD algorithm. The EMD mainly decomposes the data *y*_*t*_ into smaller signals called IMFs. An IMF is a function in which the upper and the lower envelopes are symmetric. Moreover, the number of zero-crossings and the numbers of extremes are equal or differ by one, at the most [10]. The algorithm for extracting IMFs for a given time series *y*_*t*_ is called shifting, and it consists of the following steps.

- The initial estimates for the residue are set at *r*_0_(*t*) = *y*_*t*_,  *g*_0_(*t*) = *r*_*k*−1_(*t*), and *i* = 1, and the IMF index is set at *k* = 1.
- The lower minima *I*_min⁡_*i*−1__ and the upper  *I*_max⁡_*i*−1__ envelopes of the signal are constructedusing the cubic spline method.
- The mean values *m*_*i*_ are computed by averaging the upper and lower envelopes as *m*_*i*−1_ = [*I*_max⁡_*i*−1__ + *I*_min⁡_*i*−1__]/2.
- The mean is subtracted from the original signal; that is, *g*_*i*_ = *g*_*i*−1_ − *m*_*i*−1_ and *i* = *i* + 1. Steps (i) to (iv) are repeated until *g*_*i*_ becomes an IMF. Hence, the *k*th IMF is given by IMF_*K*_ = *g*_*i*_.
- The residue is updated via  *r*_*k*_(*t*) = *r*_*k*−1_(*n*) − IMF_*K*_. This residual component is treatedas new data and subjected to the previously described process to calculate the next IMF_*K*+1_.
- The previous steps are repeated until the final residual component *r*(*x*) becomes amonotonic function. The final estimation of residue *r*(*x*) is subsequently considered.

Several methods have been presented to extract trends from a time series. Freehand and least squares methods are the commonly used techniques; the former depends on the experience of users, whereas the latter is difficult to use when the original series is very irregular [11]. The EMD is another effective method for extracting trends [6].

## 4. Local Linear Quantile (LLQ) Regression

The seminal study of Koenker and Bassett [12] introduced parametric quantile regression, which is considered an alternative to the classical regression in both parametric and nonparametric fields. Numerous models for the nonparametric approach have been introduced in statistical literature, such as the locally polynomial quantile regression by Chaudhuri [13] and the kernel methods by Koenker et al. [14]. In this paper, we adopt the LLQ regression employed by Yu and Jones [15].

Let {(*x*_*i*_, *y*_*i*_), *i* = 1 …, *n*} be bivariate observations. To estimate the *τ*th conditional quantile function of response *y*, the equation below is defined given *X* = *x*:

(1) $$\begin{array}{c} g\left(x\right)=Q_{y}\left(\tau \;|\;x\right). \end{array}$$

Let *K* be a positive symmetric unimodal kernel function, and consider the following weighted quantile regression problem:

(2) $$\begin{array}{c} \underset{\beta \in i^{2}}{\min}\overset{n}{\underset{i=1}{\sum }}w_{i}\left(x\right)\rho_{\tau }\left(y_{i}-\beta_{0}-\beta_{1}\left(x_{i}-x\right)\right), \end{array}$$

where *w*(*x*) = *k*((*x*_*i*_ − *x*)/*h*)/*h*. Once the covariate observations are centered at point, the estimate of *g*(*x*) is simply *β*_0_, which is the first component of the minimizer of (1), and determines the estimate of the slope of the function *g* at point *x*.

## 5. Bandwidth Selection

The practical performance of ${\hat{Q}}_{\alpha }(x)$ strongly depends on the selected bandwidth parameter. We adopt the strategy of Yu and Jones [15]. In sum, we employ the automatic bandwidth selection strategy for smoothing conditional quantiles as follows.

(1) We use ready-made and sophisticated methods in selecting *h*_mean_; we employ [16] which explored a “direct plugin” bandwidth selection procedure which relies on asymptotically optimal bandwidth:
  (3) h
mean
={σ2R(K)(b−a)nμ22∫{m′′(x)}2p(x)dx}1/5=C1(K){σ2(b−a)nθ22}1/5,

where and for later use we have introduced the array

(4) $$\begin{array}{c} \theta_{rs}=\int m^{r}\left(x\right)m^{s}\left(x\right)p\left(x\right)dx,\;r,s\geq 0,\;\;r+s\;\;\text{even}. \end{array}$$

Again for later use we write down the following estimator with a bandwidth *g* for  *σ* ^2^:

(5) $$\begin{array}{c} \sigma_{g}^{2}=\frac{1}{\nu }\overset{n}{\underset{i=1}{\sum }}{\left\{Y_{i}-{\hat{m}}_{g}\left(X_{i}\right)\right\}}^{2}. \end{array}$$

This is simply a normalized residual sum of squares and normalizing quantity *ν*, sometimes known as the degrees of freedom, which is given by *ν* = *n* − 2∑_*i*_ *wx*_*i*,*h*_(*X*_*i*_) + ∑_*i*,*h*_ *w* ^2^ *x*_*j*,*h*_(*X*_*i*_).

Its presented guarantees *E*(*σ*_*g*_^2^∣*X*_1_,…,*X*_*n*_) = *σ* ^2^, where *m*(*x*) is either constant or linear [17].

(2) We use *h*_*τ*_ = *h*_mean_{*τ*(1−*τ*)/*ϕ*(Φ^−1^(*τ*))^2^}^1/5^ to obtain all of the other *h*_*τ*_ *s* from *h*_mean_. *ϕ* and Φ are standard normal density and distribution functions, and *h*_mean_ is a bandwidth parameter for regression mean estimation with various existing methods. This procedure obtains identical bandwidths for the *τ* and  (1 − *τ*) quantiles.

## 6. Proposed Method

The proposed method consists of two stages that automatically decrease the boundary effects of EMD [8]. At the first stage, LLQ which is considered as an excellent boundary treatment [18] is applied to the corrupted and noisy data. The remaining series is then expected to be hidden in the residuals. At the second stage, EMD is applied to the residuals. The final estimate is the summation of the fitting estimates from LLQ and EMD. Compared with EMD, this combination obtains more accurate estimates.

This section elaborates the proposed method, EMD-LLQ. The basic idea behind the proposed method is to estimate the underlying function *f* with the sum of a set of EMD functions, *f*_EMD_, and an LLQ function, *f*_LLQ_. That is,

(6) $$\begin{array}{c} {\hat{f}}_{\text{LLQR}.\text{EMD}}={\hat{f}}_{\text{EMD}}+{\hat{f}}_{\text{LPQR}}. \end{array}$$

We estimate the two components *f*_EMD_ and  *f*_LLQ_ to obtain our proposed estimate, ${\hat{f}}_{\text{EMD}.\text{LLQ}}$, through the following steps.

(1) The LLQ is applied to the corrupt and noisy data *y*_*i*_, and the trend estimate ${\hat{f}}_{\text{LLQ}}$ is subsequently obtained.
(2) The residuals of *e*_*i*_ from LLQ, that is,  $e_{i}=y_{i}-{\hat{f}}_{\text{LLQ}}$, are determined.
(3) The EMD is applied to *e*_*i*_, given that the remaining series is expected to be hidden in the residuals. This step is accomplished by performing the following substeps: this substep is accomplished by performing algorithms (i) to (vi).
(4) The final estimate is the summation of the fitting estimates from LLQ and EMD and is as follows:
  (7) $$\begin{array}{c} {\hat{f}}_{\text{LLQ}.\text{EMD}}={\hat{f}}_{\text{EMD}}+\hat{r}\left(t\right). \end{array}$$

## 7. Experiment Analysis and Results

**Figure 1:**
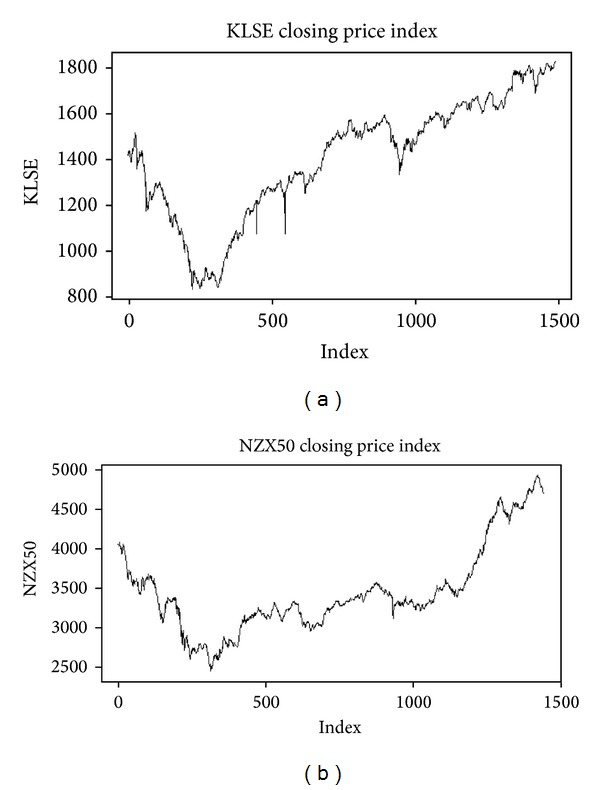
(a) and (b) KLSE price and NZX50 closing price index, respectively.

In this section, we consider the daily closing prices of two stock markets, namely, KLSE and NZX50, from December 3, 2007, to December 6, 2013, see Figure 1. The last 10, 30, and 50 days of the KLSE and NZX50 stock indices are forecasted, respectively, based on the past sequences. The selection of these two indices aims to qualitatively and culturally compare the time series of two different markets. The data used in this study are collected from the website: http://in.finance.yahoo.com/. We analyze the two indices based on the EMD-LLQ and EMD, in combination with the FFT and the Holt-Winter methods. The approach consists of several steps. First, we decompose the daily closing prices of the stock markets into a finite number of components called IMFs and one residue. Second, we select significant components by applying the FFT to each IMF. Third, we add the significant component obtained from step two to the residue to acquire the trend. Finally, we employ the Holt-Winter method for forecasting the trend.

## 8. Comparison of Forecasted Data

Two criteria are used to evaluate the forecasting performance of the different models in empirical studies. The forecasting accuracy measures employed in this study are root mean square error (RMSE), mean error (MAE), and mean absolute square error (MASE). The RMS, MA, and MASE values obtained through the EMD-LLQ, EMD, and Holt-Winter methods in each test set for the two index series are summarized in Tables 1 and 2. The results demonstrate that the proposed EMD-LLQ method is more successful in all cases in forecasting the stock closing prices than the EMD and the Holt-Winter methods.

## 9. Conclusion and Future Research

We propose an EMD-LLQ model for forecasting future prices by considering the past sequences of daily stock prices. The EMD-LLQ method is a new two-stage forecasting method that combines the EMD and LLQ algorithm. The effectiveness of the new model is analyzed by performing experiments on KLSE and NZX50 data. The capability of the EMD-LLQ for forecasting the future daily stock closing prices is better than that of LLQ, EMD, and the Holt-Winter methods. The results demonstrate that EMD-LLQ is an effective method for forecasting financial time series. Future research should improve the performance of the proposed method. We can consider several forecasting strategies by analyzing the composition of daily stock closing prices. Another possible strategy is to apply the Hilbert-Huang transform for selecting meaningful IMFs (components) instead of using the FFT.

## Figures and Tables

**Table 1:** Comparison of RMSE, MA, and MASE values for KLSE using the Holt-Winter, LLQ, EMD, and EMD-LLQ methods.

*n*.head = 10	RMSE	MAE	MASE
EMD-LLQ_*τ*(0.25)_	822.4843	468.8303	133.0985
EMD-LLQ_*τ*(0.50)_	822.2308	468.7086	129.4356
EMD-LLQ_*τ*(0.75)_	821.6983	468.3845	135.1209
EMD-LLQ_*τ*(0.95)_	822.3858	468.7594	135.0173
EMD	824.3217	470.1197	135.2341
Holt-Winters	824.0651	473.557	218.0672

**Table 2:** Comparison of RMSE, MA, and MASE values for NZX50 using the Holt-Winter, LLQ, EMD, and EMD-LLQ methods.

*n*.head = 10	RMSE	MAE	MASE
EMD-LLQ_*τ*(0.25)_	2002.658	1146.388	141.743
EMD-LLQ_*τ*(0.50)_	2003.291	1146.721	140.9419
EMD-LLQ_*τ*(0.75)_	2003.833	1146.989	146.0694
EMD-LLQ_*τ*(0.95)_	2002.921	1146.404	143.3273
EMD	2005.733	1147.129	154.8274
Holt-Winters	2010.189	1159.261	225.7123
